# Values, animal symbolism, and human-animal relationships associated to two threatened felids in Mapuche and Chilean local narratives

**DOI:** 10.1186/1746-4269-9-41

**Published:** 2013-06-13

**Authors:** Thora M Herrmann, Elke Schüttler, Pelayo Benavides, Nicolas Gálvez, Lisa Söhn, Nadja Palomo

**Affiliations:** 1 Department of Geography, Université de Montréal, CP6128 Succursale Centre-Ville, Montréal, Canada; 2 Department of Conservation Biology, Helmholtz Centre for Environmental Research GmbH – UFZ, Permoserstraße 15, Leipzig 04318, Germany; 3 Pontificia Universidad Católica de Chile, Fauna Australis Wildlife Laboratory, School of Agriculture and Forestry Sciences, Avenida del Libertador Bernardo O’Higgins 340, Santiago de Chile, Chile; 4 Pontificia Universidad Católica de Chile, Villarrica Campus and Centre for Local Development (CEDEL), O’Higgins 501, Villarrica, Chile; 5 Department of Landscape Architecture and Landscape Planning, Technische Universität München, Emil-Ramann-Straße 6, Freising 85350, Germany

**Keywords:** Human-animal interactions, Felids, Conservation psychology, Folktales, Mapuche people, Chile

## Abstract

**Background:**

The Chilean temperate rainforest has been subjected to dramatic fragmentation for agriculture and forestry exploitation. Carnivore species are particularly affected by fragmentation and the resulting resource use conflicts with humans. This study aimed at understanding values and human-animal relationships with negatively perceived threatened carnivores through the disclosure of local stories and Mapuche traditional folktales.

**Methods:**

Our mixed approach comprised the qualitative analysis of 112 stories on the kodkod cat (*Leopardus guigna*) and the puma (*Puma concolor*) collected by students (9-14 years) from 28 schools in the Araucania region within their family contexts, 10 qualitative in-depth interviews with indigenous Mapuche people, 35 traditional Mapuche legends, and the significance of naming found in ethnographic collections.

**Results:**

We revealed a quasi-extinction of traditional tales in the current knowledge pool about pumas and kodkods, local anecdotes, however, were present in significant numbers. Values associated to both felids were manifold, ranging from negativistic to positive values. While pumas played an important role in people’s spirituality, negative mythological connotations persisted in kodkod stories. Four prominent relationships were derived: (1) Both felids represent threats to livestock, pumas even to life, (2) both felids are symbols for upcoming negative events, (3) pumas are spiritual creatures, and (4) kodkods are threatened by humans. Recommendations are provided for stimulating new ways of perceiving unpopular and threatened carnivores among those who live in vicinity to them.

## Background

The on-going loss of habitat, alteration and fragmentation represent severe threats to biodiversity globally [1]. Carnivores, among them many species of wild cats, which generally require large areas over which to forage, are particularly affected by land use change and the resulting loss of prey species and habitat [2]. As part of the on-going loss of habitat, people encroach into natural habitats provoking competition between humans and wildlife. Conflicts arise particularly among carnivores when they are perceived to harm livestock, lifestyles or even life [3]. Inskip & Zimmermann [4] reviewed the global patterns of human-felid conflicts and found that 75% of the world’s 37 felid species are involved in livestock depredation, attacks on humans or are killed in retaliation. Conflict between people and felids is one of the most urgent wildcat conservation issues worldwide [5].

To resolve human-felid conflicts, conservation approaches must deal with the dilemma that solutions are required for both parties: people and predators [6]. It is therefore crucial to understand attitudes as such, their cultural context and dynamics. Attitudes are critical because they influence to a certain extent how people will behave [7,8]; although there are many examples from natural resources conservation that this does not hold [9]. However, as simple as this relationship might seem, attitudes towards conflictive wildlife are extremely complex and involve evolutionary, psychological, genetic, social, and cultural factors [10]. According to cognitive hierarchy people develop wildlife values from a young age [11], which are culturally constructed, identity linked, and therefore often resistant to change [12]. Values form the basis of beliefs and attitudes, and can predict behavioral intentions [13,14]. Beliefs and past experiences (e.g. positive, or non-positive interactions with wildlife) relate to factors such as age, socioeconomic status, and gender [15,16]. If we tap into people’s values, beliefs, and attitudes, we can obtain valuable insights into potential behavioural predictors (i.e. decision making) to improve conservation strategies [9].

It can be said that a tendency to use animals as symbols is present in human societies, a drive that has been described and studied by anthropology through time and from various perspectives, particularly regarding the much discussed phenomenon of “totemism” [17-22]. Even though a division between the natural world and the cultural (human) one has been established in several societies [23-27], animals have never been really detached from human activity. On the contrary, they have been intimately related to the survival of the species and therefore, they are also present at an abstract-cognitive level. As Crandall [24] asserts “the animal world is often used as a playing field for human beings to illustrate, critique, and discuss human knowledge and human experience”. Thus, moral values and human activity in general are projected on other animals, establishing metaphoric relations between them.

In most traditional societies animals play a prominent role in spirituality and cultural heritage (e.g. [28-31]). Where people and wildlife share spaces, direct and indirect contact through myths, stories, or anecdotes relates people to their animal relatives in multiple ways (e.g. [32-35]). The stories people tell indicate much about their wildlife value orientation and specific attitudes and beliefs [36,37]. The way things are culturally constructed are inextricably linked to language and discourse [19], which both play a key element in story-telling. Discourse “is a practice not just of representing the world but of signifying the world, constituting and constructing the world in meaning” ( [38]:64, cited in [39]:147). Language “remains a powerful and effective means of teaching cultural values and norms, and this is true for the values assigned to other animals and the norms associated with human use of other animals.” ( [40]:130). This relates to Robert Prus [41]:53] “Realities are created and transmitted, in the course of human interaction, through the development of shared sets of symbols”.

This paper uses a combined approach of in-depth interviews and story-telling as a means for conveying value orientations towards wildlife (e.g. [36]). Our objective is to investigate the cultural representations, spiritual beliefs, and human-animal relationships of negatively perceived threatened felids (i.e. kodkod cat and puma) through the disclosure of personal experience with these animals, of local anecdotes and Mapuche traditional folktales (*epew*), and a linguistic analysis of the provenience of their names in the traditional language Mapudungun. This will allow us to better understand the background for the development of adequate and locally situated felid conservation practices.

## The local setting

### Study area and its people

**Figure 1:**
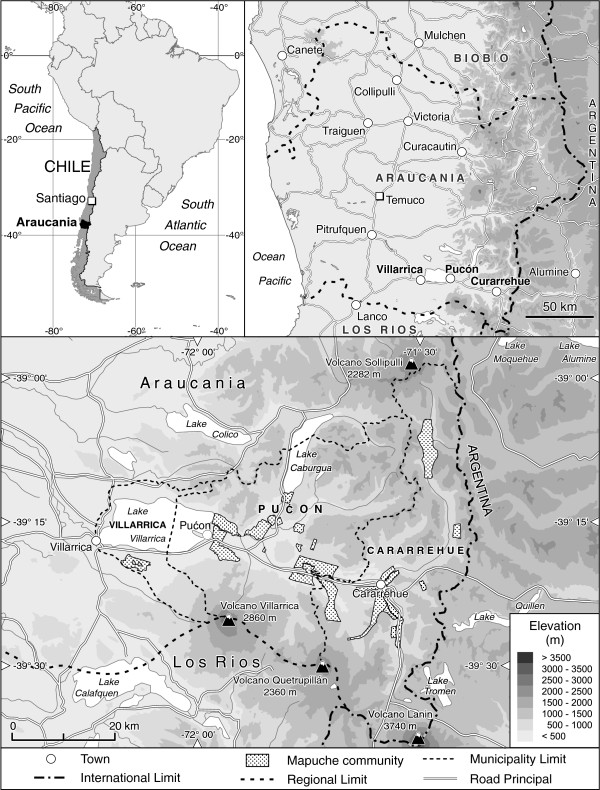
Study area.

The study was conducted in the pre-Andean zone of the Araucania region of southern Chile (Figure 1), which represents the northern limit of the South American Temperate Rainforest Eco-region (39°16´S, 71°50´W). More specifically, our study area comprised the Lake Villarica catchment which is composed of three districts: Curarrehue, Pucón and Villcarrica (43.020 inhabitants, [42]) with over 60 indigenous Mapuche communities. The Mapuche are the largest group of indigenous people in Chile (approx. 10% of the total population in Chile). While European culture has had a profound influence on their contemporary life, they still maintain many aspects of their traditional way of life [43,44]. Among the economic activities of the study area, livestock has been the main source of income in the area, but currently agriculture and animal husbandry is shifting to tourism, forestry, and fish farming. The study site is rich in wildlife, and harbours red list mammals like kodkod cats, pumas (*Puma concolor*), Southern pudus (*Pudu pudu*), and monitos del monte (*Dromiciops gliroides*). However, biodiversity is threatened through clearance and fragmentation of the lowland forest areas [45] and through pressure from heavy tourism during summer. The protected areas, represented by two national parks, one national reserve, and two private land conservation initiatives, are heavily biased towards high elevations in the Andes. This is the general case in Chile [46] and leads to insufficient protection of medium to large carnivore species offered by national parks and reserves [47].

### Studied species

The kodkod or guiña (*Leopardus guigna*) is the smallest of the South American wild cats and has one of the most restricted distributions known for felids, occupying a narrow strip within the temperate forests of south-central Chile and adjacent areas in Argentina [48]. Its distribution range coincides with the core area of indigenous Mapuche communities. Having a strong preference for dense native forest [49-51] habitat loss and fragmentation of the native temperate rainforest are considered as one of the major threats for kodkods whose population trend is decreasing [52]. A second cause for its vulnerable status is associated with retribution killing after attacks on poultry.

The puma (*Puma concolor*), also called cougar or mountain lion, is a large felid species distributed all over the Americas. It is so adaptive that it occupies a broad variety of biogeographical zones, including such extreme habitats as deserts and mountainous rangelands at high elevations [53]. Its status is listed as least concern as it is a widespread species, but considered to be declining due to habitat loss, fragmentation, poaching of their wild prey base, and retaliatory hunting [52].

## Methods

### Data collection

We selected a qualitative approach because it allows the researcher to stay in the background and let topics and concepts emerge through the participant’s own descriptions. This is particularly important when the researcher’s theoretical knowledge is limited, being the case in the field of felid-human relationships, particularly regarding the ethno-zoological view related to kodkod and puma (e.g. [54-57]). Our research combined three qualitative social research methods: (1) story collection in families as a homework by students, (2) in-depth interviews with Mapuche people, and (3) a contextual analysis of traditional Mapuche animal tales, legends, and myths with references to naming. This mix of methods should allow us to guarantee a more complete compilation of the relationships people have with wild felids in their surroundings. The story collection by students guaranteed receiving a high number of stories, but to assure receiving ethnic material also, we added empirical data from the Mapuche interviews and already published texts.

Our field research was carried out from 2009 to 2011. The stories were obtained from 21 schools located in the municipalities of Pucón and Curarrehue and 7 schools in the municipality of Villarrica. All 28 schools reflected a variety of different socio-economic status and ethnic origin (i.e. Mapuche and non-Mapuche) of the attending students. Only students between 9 and 14 years were included in the study. Through a questionnaire we first asked the students to answer a couple of closed-ended questions to identify their knowledge and perception on carnivores (Herrmann et al., in prep) before we asked them to collect stories, myths, legends, or tales in their family contexts. Those stories should focus on the kodkod cat or another carnivore in case that no story would be known about the kodkod (Pucón/Curarrehue) or about another native wildlife animal of conservation importance (Villarrica). The researchers conducting the study did not provide any information on pumas or kodkods during their school visits, neither contained the questionnaire any information, so that the pupil’s homework task was supposed to be unbiased.

10 qualitative in-depth interviews with Mapuche people (Curarrehue/Pucón) were conducted on the knowledge and the experiences people had with kodkods and pumas. The qualitative interviews consisted of semi-structured questions in which information was gathered about (1) the land use and changes in land use, (2) the knowledge and different values of the kodkod and other carnivores (e.g. puma, fox), (3) the perception and personal experience regarding the human-carnivore/kodkod/puma conflict, and (4) the socio-cultural values and role of the kodkod and other carnivores in the Mapuche culture. We used opened questions to collect stories and local anecdotes. The key informants (knowledgeable elders, chiefs) were selected upon prior informed consent at the assemblies of the indigenous Mapuche communities using a snow-ball technique.

The contextual analysis of traditional Mapuche tales was focused on the following ethnographic collections: [58-62], which are among the most important collections regarding traditional oral Mapuche records.

### Data analysis

Among the 112 collected stories on carnivores figured stories on kodkods (n=56), pumas (n=52), and foxes (n=4). The latter were excluded from the analysis due to our focus on red list felids. The stories were considered as local anecdotes due to the absence of traditional stories (expressed as myths, legends, folktales; see also [60,61]). Apart from those anecdotes, some children invented stories (n=7 on kodkods, n=9 on pumas). Those comprised personal, literary creations which probably had been explicitly made for this study. They did not represent stories that had been shared with others or handed over to others in the community/family. Therefore, all 112 collected stories will not be considered “traditional” here, although they can be considered “local” referring to their geographical origin.

We performed a content analysis [63] of the local stories, as well as interview transcriptions in order to derive an accurate reading of the kinds of settings. Different feelings and values associated to the kodkod and puma were coded by hand - deductively -, and according to the values categories (Table 1) explained hereafter. We used interpretative phenomenological analysis [64] to analyze the personal stories and the meanings of particular experiences with the two felids. This consisted in an analysis of the reflected personal experience concerned with an individual’s personal perception of the kodkod cat and the puma and with experienced events/encounters with these animals. Combining the two types of analysis facilitates both, an examination of the specific content of individual stories and an understanding of the collective perception towards these two felids. Contextual influences on story interpretation are key elements of narrative analysis grounded in the tradition of oral storytelling [65]. The contextual analysis was done using Strauss and Corbin’s [66] conditional/consequential matrix to analyze the conditions and the multiple dynamics that influence and surround the actions and interactions of participants with the two species in our study, by putting an emphasis on factors explicitly mentioned by them.

**Table 1:** **Typology for measuring cultural values and animal symbolism adapted from Kellert [**[67]**] and Vermeulen & Koziell [**[68]**]**

**Value typology**	**Related representations**
Naturalistic	1. Deep experience with nature
	2. Awareness and attentiveness, willingness to examine and discover
	3. Enhanced creativity and imagination
Ecologistic-scientific	1. Systemic study of nature
	2. Pursuit of knowledge to understand nature
	3. Cycles and system comprehension
Humanistic	1. Deep feelings of attachment to nature's components
Utilitarian (direct/indirect use)	1. Resource view (material value associated)
	2. Subsistence (or household use)
	3. Productive (or tradable use)
	4. Environment services offered to human well being
Aesthetic	1. Capacities for curiosity, imagination and creativity
	2. Recognition of order, harmony, symmetry, grace and balance
	3. Aesthetic search, real beauty, ideal and perfect
Negativistic	1. Aversive reactions to nature
	2. Destructive practices sometimes
	3. Environmental problems like pollution
Dominionistic	1. Sense of control and domination of nature
	2. Nature as a place for exercising mastery
Moralistic	1. Nature as a philosophical resource
	2. Willingness to treat nature with respect and kindness
	3. Ethical responsibility
	4. Affinity feelings
Symbolic	1. Use the sights, sounds of nature in language, and other symbolic ways
	2. Religion, spirituality, anthropomorphism
Spiritual	1. Attachment to nature through its affinity with ancestors, religion, or its role in traditional ceremonies
	2. Related to cosmovision
Cultural	1. Objects of nature that express the values of the culture superimposed on, thus linked to belongingness and identity
Existence	1. Nature existence regardless of utility humans
	2. Bequest to future generations

Diverse approaches to classify values related to species and their protection exist in the literature (e.g. [67-72]). To identify values and attitudes towards kodkod cats and pumas from the collected stories (local anectodes/invented) in this study the typologies of values by Kellert [67], and Vermeulen & Koziell [68] have been applied.

Kellert [67] proposed nine scales for measuring values towards animals (*utilitarian, naturalistic, aesthetic, ecologistic-scientific, symbolic, negativistic, humanistic, moralistic, dominionistic*). Kellert’s typology has been chosen for this study because it also includes values leading to negative attitudes. These are the *dominionistic* value (referring to the confrontation of people by wildlife and nature), and the *negativistic* value (applying to negative feelings including aversion, fear, and dislike that nature evokes). As such, we understand Kellert’s values as evaluative standards that determine preferences and attitudes (as defined by [73] in [8]), both positive and negative. Although the concept of “negative values” can be criticized (e.g. [74]), other authors also include fear and likeability when evaluating behaviours towards animals (e.g. [75-78]).

Vermeulen & Koziell [68] proposed a classification of biodiversity values by dividing them into *direct use values* (subsistence value, productive value), *indirect use values* and *non-use values* (option value, bequest value and intrinsic value). Unlike Kellert, the classification used by Vermeulen and Koziell is predominantly based on what nature can offer to humans in an ecological or economic sense (resource view). Thus, Kellert's utilitarian value is not as specific as the direct and indirect use values outlined by Vermeulen and Koziell. However, Kellert has a stronger differentiation of their non-use value. For a well-balanced and detailed representation of the values of this study we combined the two value systems. Both value systems can be interwoven and are sometimes overlapping [79]. Table 1 summarizes the adapted values typology used for data analysis in this study.

## Results

### Attitudes and values towards the kodkod cat

A total of 56 stories related to the kodkod cat were analyzed: n=49 local anecdotes and n=7 stories invented by the children themselves. Nine values of the 12 values proposed in our typology used for this analysis were found in the stories: humanistic, utilitarian, aesthetic, negativistic, dominionistic, symbolic, spiritual, moralistic, and existence. In the invented stories we found five associated values: humanistic, utilitarian, aesthetic, moralistic, and symbolic.

#### Humanistic value

In some of the invented stories the kodkod appears as a companion of humans:

*“[…] 3 years later the poor kodkod cat got sick because it had eaten a long-tailed colilargo rat and it finally died. But the owner [of the house] always will remember it as a hero because the cat gave his life to save the life of his owner. ‘Thank you for having taken care of me’ were her last words. The owner was crying a lot. A lesson learnt from this story is that kodkod cats prevent diseases.”* (Local story G38, invented by a child)

In other invented stories, the kodkod stands as symbol for friendship:

*“[…] and hundreds of mice began to appear. A big battle started between the urban cats and the mice. But the cats did not succeed in controlling the mice, when suddenly the kodkod cat appeared and started to kill the mice. Hundreds of mice died. The people in the city asked Belen where she got this magnificent cat from and she answered it [the kodkod cat] is my best friend.”* (Local story G40, invented by a child)

#### Utilitarian value

Utilitarian values associated with the kodkod cat found in the stories related either to a material use, but more often to a service the kodkod cat provided to humans, namely the killing of mice.

Some stories even expressed a feeling of respect for this service provided by the kodkod cat to humans. In some local anecdotes this emotional feeling of respect is further emphasized into admiration and proudness (i.e. the kodkod cat is perceived as a hero and friend). Thus, in some stories, one value (utilitarian) generates another value (humanistic, see local story G40, cited above):

*“[…] Yet it is an animal that fights field mice, therefore killing them is not good because it helps us to control pests.”* (Local story G17)

*“Because the kodkod eats mice, snakes, […] all those things […] that are no longer in their places of habitat and they walk around. But you should seek a balance because, if we are sharing this earth with animals, then they stand for something. If the mice invade us it is because we lack a kodkod cat nearby. Similarly, if the reptiles, the snakes, whatever it might be, invade us, it is because we lack the kodkod cat. It is the kodkod cat who consumes them.”* (Interview 5)

#### Aesthetic value

Several stories depict the kodkod cat as a beautiful animal. Especially in the invented stories:

*“[…] the father approached slowly the kodkod cat and was surprised to see a very nice animal with beautiful fur and with its extended tail and its wakeful eyes and its very hairy ears, and the father decided to take the animal home […].”* (Local story G36, invented by a child)

*“If you look at the kodkod cat you will see how cute it is […] because it has tiny little round ears.”* (Interview 14)

*“Well, the kodkod was wakeful; its fur is more beautiful than that of domestic cats. And it has small ears. The kodkod cat is really cute!”* (Interview 6)

#### Negativistic value

Over the half of the collected stories expressed negative feelings evoking displeasure and anger towards the kodkod since it kills poultry. Farmers reacted by chasing off or killing the kodkod in order to protect their livestock. We did not find feelings of fear associated with the kodkod, only repulsion towards this felid as it may endanger the farmer’s livelihood/domestic sustainability.

*“[…] it [the kodkod cat] has a bad reputation because everyone knows it to be harmful, every time it enters a henhouse it kills a lot of birds by eating only their heads, leaving the birds dead and the worst thing is that it goes away and comes back again and again until it kills all the birds.”* (Local story G17)

*Why you were hunting the kodkod? Interviewee 1: “Well, because the kodkod was doing much damage to us. They [the kodkod cats] did not allow us to raise chicken. They came every night to enter our henhouse.”* (Interview 1)

*“Well, one kills it [the kodkod cat], because it is doing damage […] Once killed one takes the leather and then sells the leather. But one kills them because they are causing damage.”* (Interview 4)

#### Dominionistic value

In none of the stories the kodkod cat appeared as an animal that people aimed to master, but as an animal to keep away from humans. Domestication of the kodkod as another type of control is seen as impossible in the local stories:

*“[He saw] a cat with four cubs; he followed this cat to catch one of her kittens. That was when his sister screamed to him: Don’t you ever grab this cat! Those are not cats, they are kodkods! This was how he discovered the kodkod cat. It is quite similar to a domestic cat. The only differences are that you can’t domesticate this animal, and it feeds on the blood of birds […].”* (Local story G7)

*“The cats were very aggressive, they would not let us touch them […] But they were always acting like this when they ate [the Interviewee mimics a cat looking rapidly from side to side]. They do not eat like those cats who relax and eat […] they are very active, very active. You gave them food and “PUM” - they disappeared out of our sight.”* (Interview 6, talking about kittens that were supposedly half kodkods)

#### Moralistic value

In some narratives, ethical conscience was reflected. The stories mention the kodkod as being an endangered species, yet this perception is not always associated with a concern about the decline of the species. In some narratives, the vulnerability of the species is merely a fact/characteristic of this animal, but not something that has to be changed. In other stories, however, an explicit motivation for moral acting existed:

*“[…] days later my aunt saw a dead kodkod and she regretted and promised to herself that she would never again beat an endangered animal.”* (Local story G18)

Interview data indicated that rarity can positively influence a moralistic attitude:

*“I really liked those things [hunting kodkods]! I liked it but now I do not anymore. […] I would not hunt it anymore because nowadays there are so few of them.”* (Interview 6)

#### Symbolic value

Very interestingly, in some local anecdotes the kodkod is an indicator of bad luck. The kodkod is seen as a sign of famine, poverty, disease, or death to the landlord:

*“[…] My neighbour said: when the kodkod enters the henhouse to eat the farm birds, this is very bad luck for the family as either poverty, famine or any kind of disease will appear that can lead to death to the owner. These are ancient beliefs […].”* (Local story G41)

Mapuche people interviewed during our fieldwork told us that the Mapudungun word *weñefe* is commonly used to describe a person who lies – referring thereby to the behavioural characteristic of the kodkod cat:

“*For us, the guiña - or wiñefe as we say - is called like that because we derived it from an animal that steals … to survive. It`s an animal that goes into the henhouse and makes a hash of things, steals, kills*.” (Interview 5)

#### Spiritual value

In the majority of the stories, the kodkod cat is represented as a being that kills by sucking the blood or by cutting the head off its prey. It seems that kodkods only eat the crop, neck, and head of a chicken, but the sucking of blood is probably more an action that we can classify as supernatural allocated to magical beasts. Further evidence to the supernatural perception of the kodkod cat is also given in a story that highlights the fact that bullets of people do not hurt them:

“*[…] my grandfather pulled a revolver and fired twice, after shooting, a kodkod cat fell from the tree. My grandfather tied its legs and paws and mounted the animal on his horse … my grandfather put the kodkod in a bag and I checked it at home to see where the animal had been shot, but I could not find any mark.”* (Local story G33)

The kodkod can be an indicator of bad luck:

*“When it [the kodkod cat] comes to a house and kills a chicken, it is a bad sign.*” (Local story G46)

#### Existence value

Every creature has a right to live, as according to one interviewee:

*“I do not agree [with kodkods being hunted], because every creature wants to live, right? Every creature wants to live and walk around the countryside, they do no damage in the house […] They want to live as anyone of us, too […].”* (Interview 14)

Others perceived the kodkod as a being of God’s creation, which also justifies its existence:

*“God brought [the kodkod] for something. If God had willed that this animal does not exist, then there would be no kodkod today."* (Interview 5)

#### **Absent values**

In none of the local stories the kodkod cat was related to naturalistic or cultural values. The ecologistic-scientific value was also absent. Yet, some stories contained a short correct biological description of this animal, thus showing the knowledge people have of the animal. The interview participants expressed their wish to understand kodkod behaviour, but the behaviour remained a mystery to the interviewee:

“*I do not know why the kodkod is hunting; if it [the kodkod] would have the opportunity to kill all chicken in a henhouse it would do so. When the puma for instance is killing a sheep or a goat it would always take its prey to a certain place […] but the kodkod does not do so, this animal would kill as much chicken as it could, but I never heard that it takes its prey to some place […] I really do not know what might be the reason for the kodkod cat to hunt?*” (Interview 27)

### Attitudes and values towards the puma

A total of 52 local anecdotes on pumas were collected (n=43 local anecdotes and n=9 invented stories). We identified nine values of the typology of 12 values (Table 1) relating to the puma: naturalistic, ecologistic-scientific, utilitarian, aesthetic, negativistic, dominionistic, moralistic, symbolic, and spiritual.

#### Naturalistic value

A naturalistic value was allocated when local anecdotes related to direct experiences with pumas:

*“[…] it was the first time that he [my dad] saw a puma, he was frightened, but now my dad sees it [the puma] as a special and beautiful animal because it [the encounter with the animal] was such a great experience.”* (Local story P30)

#### Ecologistic-scientific value

The ecologistic-scientific value was found in narratives that underlined that this felid had to kill in order to survive; that this ability was instinctive. Such a perception reflects a type of conscience of the puma ecology:

*“The two animals [pumas and pudus] are good because they act by instinct.”* (Local story P33)

Similarly, some people in the interviews talked about their wildlife observation. Others expressed their fascination and interest towards understanding the behaviour of the puma:

*“I was eager to experience; I wanted to know how the mountain lion hunts a sheep. I asked myself: "How would he do that? Would he grab the sheep by its legs? How he would grab it?" One day when I will see that the mountain lion is hunting a sheep, I'll have a look.”* (Interview 14)

#### Utilitarian value

Some interviewees referred to the utilitarian value associated to the puma, especially to a service the puma provides to humans, namely the killing of harmful animals.

*“Based on their food they [the puma and kodkod] will have to control some animal [populations]. So I guess for example […] thanks to the puma there are not so many wild boars,[…], or that there are not so many harmful bird because those are eventually eaten by kodkod cats.”* (Interview 27)

#### Aesthetic value

The local anecdotes frequently revealed the puma to be an agile animal but also as a brave, wild, fast, beautiful, and special animal:

*“[…] Mom said that the puma was beautiful and that he had a beautiful pair of claws […].”* (Local story P38)

#### Negativistic value

The puma is associated to a negativistic value, i.e. a predator that not only chases and kills farm animals but could also attack and even kill humans. There is a clear expression of emotional feeling related to fear:

*“[…] During winter, on days of heavy rain when the water flooded the marshes, this family owned a house near a creek and about 5 pm, a terrible mountain lion jumped onto the roof of the house; the family cried for help, but nobody could help them. Then they started screaming, beating on pots, beating on the walls until the puma jumped from where he was and disappeared through the trees surrounding the house there. The father went to the nearest neighbours to tell them what had happened.”* (Local story P19)

*“One afternoon in April, when the woman as usual went to the river with two jugs of 5 liters to fetch water, at night when the husband returned from his work he did not find the woman at home. He waited a few minutes since he knew she was fetching for water because the jugs were not in the house. Then he went to the river and saw the two jars without water and scattered on the ground, and traces of blood. He followed the traces and found the body of his wife destroyed. He returned home and […] prepared a large knife, a machete … and with it he returned to the body of his wife. There he stayed […], waited until 4 am, when the puma came. Full of rage, he pierced the knife into the heart of the wild animal.”* (Local story P47)

*“Oh yes, they [the elders] would teach you, tell you which animal is dangerous. As children, we said for example one must be careful with the mountain lion… with the wild boar, because these animals can attack humans … so we have to fear them.”* (Interview 26)

#### Dominionistic value

In a number of stories people fought with a puma and triumphed over it (see also story P47, above):

*“In Licanray many people still remember him [Carmelo H.] as the only man who had the courage to kill a puma.”* (Local story P49)

#### Moralistic value

In some stories, people expressed the wish that pumas should not be hunted:

*“I think that they should not have hunted this puma because it [the female puma] was only teaching her cubs how to hunt for surviving, as any mother would do.”* (Local story P23)

#### Symbolic value

In several local anecdotes, the puma is represented as a symbol for long life and immortality. Humans can reach these traits when eating the meat of a puma:

*“They killed this animal [the puma] and decided to eat it. Afterwards each person went home. The next day at dawn when they looked in the mirror they saw that they had claws, sharp teeth, much more hair as usual but a gentle skin and after some days they started walking on all fours. At the end they became the animal that they killed - this animal is called a puma. That is why the local people say that pumas can be human beings, but only at night, and they [pumas] never die.”* (Local story P34)

*“The elders tell that the one, who eats puma meat, never dies and lives many years.”* (Local story P49)

*“It [the puma] is a very lonely animal, very powerful. They live in hidden places.”* (Interview 31)

Often the puma stands for the king of the mountains:

“*There is a legend about a mysterious puma who liked to eat fresh meat; he was the king of all the mountains and more powerful than most of all carnivorous and wild animals*.” (Local story P17)

#### Spiritual value

Some stories portray the belief that pumas could walk like humans, and that they are invincible knights:

*“Many years ago in the Middle Age, it is said that pumas were able to walk like humans, they were gentlemen. At that time they were invincible, they were the companions of the King in the war […].”* (Local story P50, invented by a child)

The spiritual value is strongly related to feelings of respect and admiration for the abilities of this animal:

*“They name it [the puma] pangi, trapial or ñelmapu[Mapudungun words] … well he has several names. In fact people never call him by his real name because he is a much feared animal. When you named it by its name, the puma would come to you. So people used nicknames, such as trapial or ñelmapu, never … they called this animal by his real name, which is pangi.”* (Interview 31)

Just as for the kodkod, we found that the puma can be an indicator of bad luck or a sign that someone shall die:

*“My grandfather said that it is very bad when a puma is close to a house; a family member would die.”* (Local story P42)

Several people referred to the special powers the puma holds:

*“Well, when one goes up into the mountains, one has to […] ask it [ask the puma for permission]; he protects us also because he is like the owner of the mountains.”* (Interview 31)

*“The fat of the mountain lion [the puma] played an important role […] if one were a cyclist, and had a ball of fat from the lion, one always would win … before you run, you cut a career (way) out there and no one will run faster than you, you would win the race … and they did the same with the horses, no other horse would be faster. […] Who has ever won a fight with the lion? No one, one pays the fight with a puma only with death.”* (Interview 14)

#### Absent values

In none of the local stories the puma was related to cultural, humanistic, or existence values.

### Kodkod and puma symbolism in traditional Mapuche stories

Story-telling forms an integral part of the Mapuche culture. Yet, the kodkod cat does never appear as a main character in recorded traditional Mapuche stories. In two of the 22 stories collected by Pino [61] it only appeared as a side character, and it is absent in the 13 animal stories collected by Kuramochi [60].

However, in popular Chilean culture the second name also attributed to the cat - guiña - refers to a person who is a fast and effective thief, possibly relating to the word *wiñen* “to steal” or *weñefe* “thief”. Thus, the kodkod cat seems to be connected to their occasional attacks on chicken and thus becomes a symbol of a thief. Interestingly enough, most people who use this word to refer to a thief rarely know that the kodkod cat exists. According to Villagrán et al. [80], the word guiña is derived from the Mapudungun word *wiñamn* "to carry", "transfer" or "change of place". One could advance the hypothesis that this may reflect one of the animal's behavioural characteristics, alluding to the act of carrying preys, like poultry. There is however no evidence on this matter.

According to Mora Penroz [81], the Mapudungun word *Kona* “warrior” is composed of two particles *ko* “enter something” and *naln* “fighting aggressively”. Mora Penroz [81] suggests that *ko* might come from *kod-kod* the Mapudungun name for *Leopardus guigna*. According to this author, the reduplicated syllable probably emphasizes an outstanding quality of this animal (e.g. starting an attack with the head up like a battering ram). The linguist Havestadt, in the nineteenth century, collected the word *konalu* meaning “offenses against the reverence due”/“acting wild” which according to Mora Penroz [81] might refer to the way the wild kodkod cat is behaving. Accordingly, the family name *Kona* evokes a distant and vague feline-like behaviour. Further research is necessary to better understand the etymology of the word.

The puma or *pangui* in Mapudungun plays a significant role in Mapuche traditional narratives. He is the main or a central character in 5 of the 22 stories collected by Pino [61], and in two of 13 animal stories collected by Kuramochi [60]. The puma represents an animal of prestige in Mapuche culture; warrior initiations included the introduction of particles of a puma bone [81]. A reference to the puma is part of many Mapuche names, patronymes and toponymes (e.g. *Painepan*, *Panguipulli*, “the spirit of the puma”, *Panguilef*, *Lefpan*, “the running puma”, [82]). The Mapuche also called the Mountain lion *Futamaye* “Great Uncle” which shows its relationship to humans ( [83]: 280). The qualities attributed to this felid, such as being a strong, vigilant warrior, performing agile attacks, are underlined in many historical Mapuche stories [58,60,61]. Foerster [84] in his structural analysis of Mapuche stories argues that in those where puma and foxes are confronted, the puma represent the paternal uncle whereas the fox stands for his nephew.

Very possibly, the name *pangui* is related to the Mapudungun word *pane* “semen”, thus linking the male sperm liquid to the high (holy) virtues of the feline movement [81].

## Discussion

**Figure 2:**
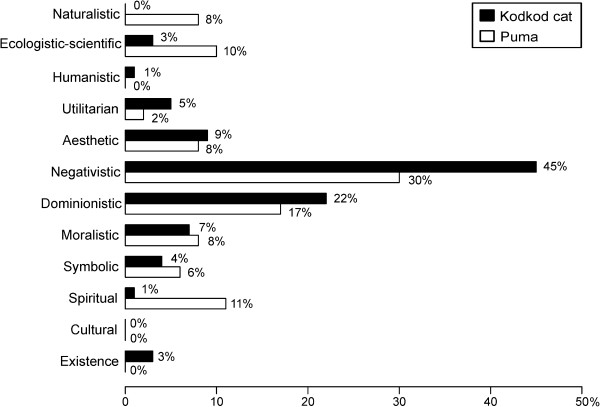
Values represented in local anecdotes (n=76 values in 49 local anecdotes for kodkod cats, n=63 values in 43 anecdotes for pumas).

Our results disclosed a variety of values among two endangered felid species (Figure 2) giving insights into positive, negative and absent associations (summary in Table 2). These associations can be broadly divided into two distinct perspectives: one seeing the felids as a real threat to livestock and humans, the other considering these two carnivores as symbolic/spiritual identities. We agree with Álvares et al. [85] in that these two perspectives are closely connected, suggesting that the human-felid conflict is entrenched in both the carnivore’s role as a predator on domestic animals and livestock, but also in its mythical and spiritual character. In the following we try to get a deeper understanding of the human-animal relationships that emerge from the value and symbolic associations.

**Table 2:** **Overview of the values (adaped from [**[67]**,**[68]**]) associated to kodkod cat and puma.**

**Values table 1**	**Kodkod cat**	**Puma**
Naturalistic		✓
Ecologistic-scientific		✓
Humanistic	✓	
Utilitarian	✓	✓
Aesthetic	✓	✓
Moralistic	✓	✓
Cultural		
Existence	✓	
Negativistic	✓	✓
Dominionistic	✓	✓
Symbolic	✓	✓
Spiritual	✓	✓

### Both felids represent threats to livestock, pumas even to life

Wild carnivores frequently engender negative attitudes among people worldwide where they prey upon domestic animals. This economic conflict is well documented, for example for snow leopards [86], wolfs [87-89], and wild cat predators such as jaguars and pumas [54,55]. In our study, the majority of the stories regarding kodkods contained negativistic and dominionistic values, mainly allocated to the animal due to its characteristic poultry killing. Poultry killing by these small wild cats is deeply anchored in the cultural history of rural households ( [90]:333), although nowadays comparably few incidents really happen (Schüttler et al., in prep). The puma was also described as a severe predator of farm animals (mostly sheep). However, beliefs about pumas seem to play an even greater role within the conflict than do economic concerns (see also [91]): It is usually blamed for predation even if other species (e.g. domestic dogs) are involved (Murphy et al. unpublished data). Perhaps the “bold” behaviour described in the narratives governs how a predation event is perceived and inhibits all other plausible explanations.

Our stories also described pumas as predators on human lives. This finding is in line with the literature where a number of large wild felid species, such as tigers, lions, and leopards, in historical accounts have been attributed to people-eating incidents [92-96]. While kodkods do not represent a direct danger to humans, puma stories expressed fear of being killed by them. However, in Chile records of puma attacks on people are extremely rare with only one fatal attack having occurred in modern times ( [97] in [91]). Thus, the clear expression of fear is unlikely to arise from stories or events derived from physical contact with the feline, but rather from mind construction of the consequences (given the nature of the animal, the puma seems to be perceived more as a bold animal). Conforti & De Azevedo [54] found similar results regarding cultural manifestations related to the jaguar in south Brazil. Here, cases of people killed by jaguars occurred rarely and only in a poaching situation; hence, the authors concluded that negative perceptions seemed not to be based on the real risk the species may represent to people, but rather the perceived risk.

### Both felids are symbols for upcoming negative events

Several scholars have shown that perception of carnivores by people, who have a long coexistence with carnivores, are often based on ancient, religious, symbolic beliefs, and ancestrally rooted practices that go beyond the simple view of it as a threat to livestock [85,98]. For example, Álvares et al. [85] illustrated how local people perceive the wolf from a double and antagonistic point of view: as a totemic and benign animal, a relic from pagan religions of Celtic influence, and as a diabolic creature, legacy of the Christian mentality of the Middle Ages. Trout [99] asserts that human beings have sublimated primeval fears into mythical narratives and symbols. A long history as hominid preys, particularly of big cats, has generated the adaptative biological disposition to fear big felids and to initially associate their traits (and the ones of other predators such as crocodiles, wolves, etc.) to danger and menace. This in turn has been extensively used in the construction of the monsters that emerge in narratives and other products of popular culture, to this day.

Both felids in our study were associated with beliefs that Gods or dark forces give them supernatural power (symbolic, spiritual values). In this vein, kodkods and pumas were described as indicators for bad luck: it was taken as a bad omen when the felids would come near the person’s home. This is also known from birds in the Mapuche culture: Faron [100] described how the most frequently observed supernatural agents were malevolent night birds, seen or heard near one's house. Likewise, nocturnal birds with eerie calls (e.g. owls) were strongly associated with witches and bad luck.

The stories revealed that kodkod cats and pumas were - even more drastically - signs for an upcoming death in the family. These negative perceptions are common among landholders that co-exist with wild carnivores that attack livestock (e.g. [3,101,102]), particularly when experiencing an attack to the means of subsistence in the case of socioeconomic vulnerable families [103].

Having a look at the people’s concept of space can help to understand the reasons behind these negative connotations. Structural anthropology analysis [20] underlines the loss of balance between the expected order and the understandable limits of space: the cultured-human world versus the wild-animal world beyond (e.g. in the forest, mountains, dark areas, night). The wild animal would be an invader and “matter out of place”. This notion of animals “out of place” follows the logic of Mary Douglas’s statement: “dirt is matter out of place” ( [27]:450). In this sense, the author captures the strength of spatial organization as a vital structuring principle of general order. In his view, an “unaffiliated animal” which leaves its habitat and penetrates another one of primary value to people will be subject of strong attitudes by the affected community. The typical ones would be its consideration as a food taboo or as “a bad omen or inauspicious sign” ( [27]:450).

Kodkods are extremely inconspicuous cryptic animals and this fact might be the reason of negative cultural constructs on such animals. They are a “phantom to human observers” as Maehr [104] accurately described the Florida panther due to its elusive habits and the difficulty of sighting one in the wild. As our results indicate, the idiom associated to the kodkod referring to “being a guiña” as being a thief was frequently encountered, not only in the local stories, but also in the linguistic material. This is of relevance as such cultural constructs convey deeper attitudes towards the animal itself. Here we can link again to Tambiah’s study (1969) on animal symbolism, who by drawing a line between wild and domestic animals, notes that less known wild animals are used by the villagers to represent the social deviant, in this case a criminal figure: the thief.

### Pumas are spiritual creatures

Stories on pumas contained various dimensions of positive relationships, even including positive symbolic (long life, immortality) and spiritual (invincible, powerful animal) orientations that were absent in kodkod stories. As a precondition to accepting human presence in the puma’s authorized territory, humans have to acknowledge his authority (e.g. the puma spirit as the guardian of the mountains) and be obedient. Only then, the puma - which is characterized as a potent but merciful lord - will protect people. Altogether, the spiritual image of the puma seems to be that of a superior creature which is neither good nor bad, but that should be respected for the higher power it has and from which humans cannot escape.

### Kodkods are threatened by humans

Our data revealed positive values related to kodkods. These were a result of a growing environmental conscience and the access to information outside the family context that point to the biological function (i.e. controller of rodents, utilitarian value), the endangered status and the environmental situation (i.e. deforestation of native forest, moralistic value). Interestingly, positive values on kodkods particularly appeared in stories invented by the children themselves. Those stories also lacked a reference to the felid-livestock conflict. This finding is in line with studies that have shown an intergenerational dynamic with a trend away from domination wildlife value orientations (prioritization of human well-being over wildlife) among older people versus a more mutualistic oriented view on wildlife (animals as part of a large family deserving of rights and care) among younger generations [105]. This probably results in a shift of traditional utilitarian wildlife value orientations towards a more protection-oriented worldview [106].

## Conclusions

Understanding and managing wildlife is also about understanding and managing societies. As our results have shown, local stories can be used to reveal human-animal relationships, and to illuminate some of the underlying causes of human-wildlife conflict. Human dimensions are offering social-science information into the decisions about wildlife management, and thus have a high potential to contribute to wildlife conservation efforts. Wildlife managers, conservation biologists, and local communities increasingly recognize that the success of conservation initiatives will axis on an interdisciplinary approach and require to reflect the sociocultural, economic, and ecological components of wildlife management [107]. By determining human values and attitudes towards negatively perceived carnivores, as done in this study on two endangered felids, we first identify four main barriers to conservation and then provide recommendations on management and educational programs, general and on-site.

The four main barriers to conservation identified for our studied felid species are most probably also valid for other conflictive carnivore species: (1) *fear towards the animal* which can lead to less acceptance of its presence or even reduce the willingness to protect it (e.g. [108,109]), (2) *inconspicuousness of and missing contact possibility with the animal* which favours the willingness to protect aposematic animals over cryptic animals (e.g. [110]), (3) a *diminished or missing cultural dimension of the animal* which might provoke less identification with the animal, and (4) a *contradictory relationship towards the animal* meaning that people have mixed sentiments (positive and negative at once) towards the animal, where it is not clear which values finally govern (e.g. [111]). Knowing about these barriers to conservation, it is possible to respect them in conservation programs. Education curricula should thus transmit objective information on biology and ecology of the carnivore to reduce fear and include multimedia to familiarize people with less visible animals. Bio-cultural conservation actions could revitalize the dispersion of traditional stories to retrieve cultural relationships with the animal.

Our study offers several implications for bio-cultural conservation education on-site: (1) The elaboration of bio-cultural books for a broader public (e.g. bilingual in Spanish-Mapudungun) containing narratives and illustrations of kodkods, pumas, and other threatened carnivores would offer a fascinating view into the world of animals as understood by the Mapuche and local communities, and could serve as a creative medium for advocacy for bio-cultural conservation. (2) The findings of our study are also of value for environmental education material for school curricula focusing for example on the importance of predator-prey relationships and the biological control of mice in the case of kodkod cats. (3) The more proactive strategies to human-carnivore conflict are designed and applied, the better we may succeed to create a positive and stable coexistence with carnivores in the vicinity of Chilean temperate forests.

## Competing interests

The authors declare that they have no competing interests.

## Authors’ contribution

TMH conceived of the study, designed and carried out questionnaires among the schools in Pucon and Currarehue region, analyzed the tales, and drafted the manuscript. ES participated in the design of questionnaires, carried out questionnaires in Currarehue schools, participated in coordination, participated in data analysis and helped to draft the manuscript. PB carried out questionnaires in schools in Villarrica and interviews with local Mapuche people and helped to draft the manuscript. NG participated in carrying out and analyzed questionnaires in schools in Villarrica and helped to draft the manuscript. LS carried out and analyzed interviews with local Mapuche people and helped to draft the manuscript. NP performed statistical analysis. All authors read and approved the final manuscript.
